# Evaluation of a New IFN-γ Release Assay for Rapid Diagnosis of Active Tuberculosis in a High-Incidence Setting

**DOI:** 10.3389/fcimb.2017.00117

**Published:** 2017-04-11

**Authors:** Gen Li, Feng Li, Hui-Min Zhao, Han-Li Wen, Hai-Cong Li, Chun-Ling Li, Ping Ji, Peng Xu, Kang Wu, Zhi-Dong Hu, Shui-Hua Lu, Douglas B. Lowrie, Jian-Xin Lv, Xiao-Yong Fan

**Affiliations:** ^1^Shanghai Public Health Clinical Center, Key Laboratory of Medical Molecular Virology of MOE/MOH, Fudan UniversityShanghai, China; ^2^School of Laboratory Medicine and Life Science, Wenzhou Medical UniversityWenzhou, China; ^3^TB Center, Shanghai Emerging and Re-emerging Infectious Disease Institute, Fudan UniersityShanghai, China

**Keywords:** tuberculosis, immunodiagnosis, IFN-γ release assays, ELISPOT

## Abstract

Blood-based interferon-gamma (IFN-γ) release assays (IGRAs) have been proven to be useful in the diagnosis of *Mycobacterium tuberculosis* (*Mtb*) infection. However, IGRAs have not been recommended for clinical practice in most low-income settings due to cost-intensive limitations and shortage of clinical data available. The established T-SPOT. *TB* assay containing *Mtb*-specific antigens ESAT-6 and CFP10 are widely used for immunodiagonsis of *Mtb* infection, but the high cost is one of the restricting factors against its clinical application in the developing countries. More recently, a cost-saving IGRA assay, TS-SPOT, was approved in China. This new assay contains an additional antigen Rv3615c. Rv3615c contains broadly recognized CD4^+^ and CD8^+^ epitopes, and T-cell responses to Rv3615c are as specific for *Mtb* infection as the responses to ESAT-6 and CFP10 in both *Mtb*-infected humans and *M. bovis*-infected cattle. Therefore, we assessed the likely effect of inclusion of Rv3615c as stimulus besides ESAT-6 and CFP10 in an IGRA assay and evaluated the performance of TS-SPOT for diagnosis of *Mtb* infection and active TB compared with T-SPOT.*TB*. We tested 155 active TB patients, 90 non-TB lung disease patients, and 55 healthy individuals. The results presented an improved positive rate for diagnosis of active TB and *Mtb* infection, that could be attributable to inclusion of Rv3615c in the mixture of stimulatory antigens. The diagnostic efficiency of TS-SPOT assay for active TB was as follows: sensitivity 80.00%, specificity 83.45%, positive predictive value (PPV) 83.78%, negative predictive value (NPV) 83.45%, positive likelihood ratio (LR+) 4.83, and negative likelihood ratio (LR−) 0.24. The results were similar to those of T-SPOT.*TB*, with an excellent agreement (κ = 0.91, 95% CI: 0.85–0.95) being observed between these two assays. The sensitivities of the TS-SPOT assay varied for patients with different forms of active TB, with the highest sensitivity for patients with culture-positive pulmonary TB (92.16%) and the lowest for those with tuberculosis meningitis (50.00%). Taken together, the current evidence indicates that this new TS-SPOT assay is a useful adjunct to the current tests for rapid diagnosis of active TB and *Mtb* infection in low-income and high-incidence settings due to its characteristics of cost-effectiveness and high-quality.

## Introduction

Tuberculosis (TB), the disease caused by *Mycobacterium tuberculosis* (*Mtb*), still remains a major global public health concern. According to the updated WHO global TB report, approximately one-third of the world's populations are infected asymptomatically with *Mtb*, and about 5–10% of these people develop active TB. In 2015, there were an estimated 10.4 million new TB cases and 1.4 million TB deaths worldwide, and an additional 0.4 million deaths resulting from TB disease among people co-infected with HIV (WHO, 2016). Although the number of TB deaths dropped by 22% during the period of 2000–2015, TB remained one of the top 10 causes of death from any cause worldwide in 2015 (WHO, 2016). The results of the fifth Chinese national TB epidemiological survey in 2010 showed that the TB prevalence was 469/100,000 among population over 15 years old, and 4.99 million active TB cases were estimated across the country (The Office of the Fifth National TB Epidemiological Survey, 2012).

In China, diagnosis of TB is usually based on a combination of regular doctor inquiry, clinical presentation, radiological and pathological changes, and bacteriological findings of acid-fast bacilli (AFB). However, the smear microscopy for AFB has a low sensitivity as only 44% of all new adult cases and 15–20% of childhood cases are identified by the presence of AFB in sputum smears (Pai and O'Brien, 2008). The gold standard for the definite diagnosis of TB is the detection of *Mtb* by bacterial culture, which usually takes >1 month to provide a diagnostic end-point and delays the treatment initiation (Richeldi, 2006). Moreover, diagnosis and treatment decisions may be difficult in cases with clinical suspicion of TB and negative AFB sputum smears. Being rapid and easy to apply, tuberculin skin test (TST) has been used as an immunodiagnostic tool to support the physician's decision process for decades, but it suffers from poor specificity due to the cross-reaction of non-tuberculosis mycobacteria (NTM) or Bacillus Calmette-Guerin (BCG) vaccination (Richeldi, 2006), especially in developing countries with high TB prevalence.

In recent years, blood-based *in vitro* interferon (IFN)-γ release assays (IGRAs) have been developed as alternatives to TST for the rapid immunodiagnosis of *Mtb* infection. Both TST and IGRAs detect the presence of persistent *Mtb*-specific T-cell responses and represent indirect markers for past or present infection (Young et al., 2009). IGRAs measure IFN-γ secretion after *in vitro* stimulation of whole blood or peripheral blood mononuclear cells (PBMCs) with antigens (ESAT-6 and CFP10). These antigens are encoded in the region of difference-1 (RD1), a portion of *Mtb* genome absent among all BCG strains and most NTM species (Rangaka et al., 2012). Currently, two commercial systems are available: the QuantiFERON-TB Gold in-tube assay (QFT-GIT; Cellestis, Carnegie, Australia) that also includes a third antigen TB7.7 and measures IFN-γ using an ELISA method; the TSPOT.*TB* assay (Oxford Immunotec, Abingdon, UK) that quantitates IFN-γ-producing cells with the enzyme-linked immunospot (ELISPOT) technique.

Compared to TST, IGRAs are as sensitive and more specific for detecting latent tuberculosis infection (LTBI; Pai et al., 2008) and have better correlation with gradient of *Mtb* exposure (Ewer et al., 2003; Hill et al., 2005; Diel et al., 2006). However, neither IGRAs performed on blood nor TST appear to be able to distinguish between individuals with LTBI and active TB (ATB; Kang et al., 2007; Pai et al., 2008). Although these tests were primarily developed for the diagnosis of latent TB, clinicians have also been searching for improved diagnostic tools and explored IGRAs for the immunodiagnosis of active TB (Kang et al., 2007; Nishimura et al., 2008; Winqvist et al., 2009). In 2010, the USA Centers for Disease Control and Prevention (CDC) updated their guidelines for testing for TB infection, and concluded that IGRAs may be used instead of TST in all situations in which CDC recommends TST as an aid to diagnosis of *Mtb* infection (Mazurek et al., 2010). Recently, IGRAs have also been evaluated for the diagnosis of active TB directly on extrasanguinous fluids from sites of infection, including bronchoalveolar lavage fluid (BALF; Nishimura et al., 2008; Winqvist et al., 2009), pleural effusion (PE; Metcalfe et al., 2010) and cerebrospinal fluid (CSF; Thomas et al., 2008).

Nevertheless, in most developing countries including China, the clinical utilization of IGRAs is not recommended due to insufficient evidence of their performance in high TB burden settings (Metcalfe et al., 2011). Additionally, the high cost of QFT-GIT and T-SPOT.*TB* is one of the restricting factors against their clinical application in developing countries (Steffen et al., 2013). More recently, a domestic IGRA named TS-SPOT (Tongsheng Biotech, Beijing, China) was licensed by the China Food and Drug Administration (CFDA). This test includes a third *Mtb*-specific antigen Rv3615c as stimulus besides ESAT-6 and CFP10 and saves nearly half of cost compared to the two widely used IGRAs systems mentioned above. Antigen Rv3615c (Esx-1 substrate protein C, EspC), encoded outside RD1, is similar in size and sequence homology to ESAT-6 and CFP10 (MacGurn et al., 2005) and has high specificity to confer strong potential for T-cell-based immunodiagnosis (Sidders et al., 2008; Millington et al., 2011) and vaccine development (Kong et al., 2014; Teng et al., 2015). Rv3615c contains broadly recognized CD4^+^ and CD8^+^ epitopes and T-cell responses to Rv3615c are as specific for *Mtb* infection as the responses to ESAT-6 and CFP10 in both *Mtb*-infected humans (Millington et al., 2011) and *M. bovis*-infected cattle (Sidders et al., 2008). In this study, we aimed to determine whether inclusion of Rv3615c as an additional stimulus in the IGRA assay improved the diagnosis efficiency on *Mtb* infection compared with T-SPOT.*TB* in populations at various levels of risk in China. We also assessed whether this newly licensed TS-SPOT assay could be used for the diagnosis of active TB. A cheap assay with high quality will definitely facilitate the widespread clinical practice of IGRAs leading to the accumulation of more clinical data and clarification of its clinical value for ATB diagnosis in developing countries with high TB burden.

## Materials and methods

### Clinical trial design and participants

In this prospective clinical study, a total of 307 participants were recruited in Shanghai Public Health Clinical Center (SPHCC, Shanghai, China) from January to December 2015. Characteristics of all participants are shown in Table 1. Study protocols were approved by the Institutional Review Board of SPHCC, and written informed consent was obtained from all participants. All the participants were HIV-negative and had BCG vaccination in early childhood or during adolescence.

**Table 1:** **Characteristics of different groups of subjects in the study**.

**Characteristics**	**ATB patients (*n* = 155)**		**NATB controls (*n* = 145)**	
	**PTB (*n* = 118)**	**EPTB (*n* = 37)**	**Non-TB (*n* = 90)**	**HC (*n* = 55)**
Age, years (mean± SD)	44.29 ± 20.29	30.24 ± 20.11	42.62 ± 22.54	28.47 ± 6.76
≤25 (*n*)	25	14	21	24
26–35 (*n*)	23	8	10	18
36–45 (*n*)	13	5	16	12
46–55 (*n*)	14	4	14	1
56–65 (*n*)	21	3	13	0
>65 (*n*)	22	3	16	0
Gender (male: female)	76: 42	19: 18	52: 38	41: 14
BCG vaccinated (%)	100	100	100	100
Diabets mellitus (*n*)	9	1	1	0
Liver disease (*n*)	5	1	0	0
Autoimmune diseases (*n*)	2	0	0	0
Chronic renal failure (*n*)	1	1	0	0

**Figure 1:**
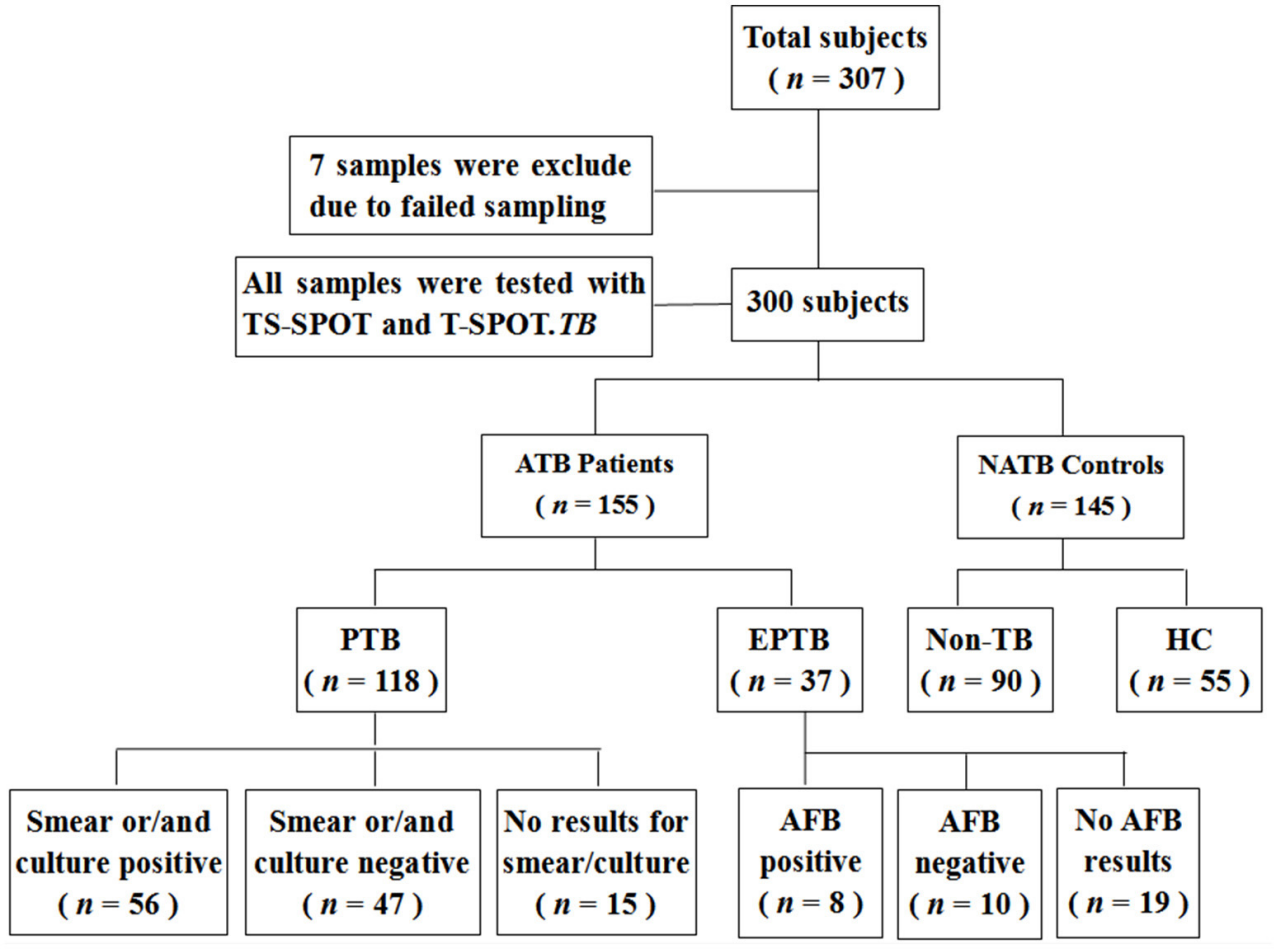
**Flowchart of the clinical validation study**.

As for the suspected TB cases, the diagnosis of active tuberculosis (ATB) patients was made on the basis of all the clinical, radiological, microbiological, and pathological information collected after recruitment and response to anti-TB therapy for at least 3 months. ATB subjects were further classified into cases of pulmonary tuberculosis (PTB) and extra-pulmonary tuberculosis (EPTB) subgroups. Recruited patients were classified as Non-TB and therefore excluded from ATB when they had an established alternative diagnosis, which referred to lung cancer, pneumonia, chronic bronchitis, and bronchiectasis that are easily confused with TB. For comparison, 55 healthy individuals with normal chest radiographs, no known history of TB infection and no symptoms of active TB, were also recruited. Ultimately, 155 ATB including 118 PTB and 37 EPTB and 145 NATB controls including 90 Non-TB pulmonary disease patients and 55 healthy individuals were tested with both TS-SPOT and T-SPOT.*TB* assays (Figure 1).

### *Ex vivo* IFN-γ ELISPOT assay

The fresh blood samples were collected before treatment or within 7 days at the beginning of treatment from participants and PBMCs were isolated from a whole blood sample by centrifugation over Ficoll density gradient (TBD Science, Tianjing, China) in a bio-safety level 3 (BSL-3) lab. Cells were re-suspended in AIM-V medium (Gibco, ThermoFisher, USA) and divided into two aliquots. A total of 2.5 × 10^5^ cells/well were seeded in 96-well plates precoated with anti-IFN-γ capture monoclonal antibody, and *ex vivo* ELISPOT assays were performed according to the manufacturer's operating instruction. Briefly, cells were incubated for 18–20 h at 37°C, 5% CO_2_ with the different *Mtb*-specific antigens (one peptide pool including ESAT-6, CFP10, and Rv3615c peptides for TS-SPOT, two separate peptide pools including ESAT-6 or CFP10 peptides for T-SPOT.*TB*) to stimulate IFN-γ secretion by the effector T cells. PBMCs in medium alone or stimulated with phytohemagglutinin (PHA) at 2.5 μg/ml were used as negative or positive controls, respectively. Biotinylated anti-IFN-γ detection monoclonal antibody was then added for 4 h, and followed by the addition of streptavidin-enzyme conjugate for 1 h. After a washing step, the chromogenic substrate was added and the individual spots were counted by use of an automated image analysis system, ELISPOT reader (Champspot III; Sage Creation Science, Beijing, China).

The result of TS-SPOT was considered to be positive if either Panel Test (containing ESAT-6/CFP-10/Rv3615c peptides pool) showed at least six spot-forming cells (SFCs) more than the negative control when the negative control ≤5 SFCs; or if the number of spots in Panel Test was at least double the number in the negative control when the negative control >5 SFCs. For the Oxford T-SPOT.*TB* assay, the result was positive if either of the two panels or both of them showed at least six SFCs more than the negative control when the negative control ≤5 SFCs; or if the number of spots in the Panel Test was at least double the number in the negative control when the negative control >5 SFCs.

### Radiographic image examination and analysis

Chest radiographs of PTB patients were reviewed by two experienced, board-certified radiologists who were blinded to the TS-SPOT results. Images were assessed for the presence and distribution of parenchymal abnormalities consistent with infiltrates and/or cavities to determine the extent and severity of disease. Pulmonary disease was defined as severe when lesions characterized by lobe infiltrate, pleural effusions, and cavities involved two or more lobes in one or both lungs (Geng et al., 2005).

### Detection of *Mtb* DNA in sputum by real-time PCR

*Mtb* DNA detection was routinely performed with aliquots of NALC-NaOH treated sputum specimens as a method to amplify a 254-bp fragment of *Mtb* IS6110 gene for TB diagnosis by using a real-time PCR kit (Daan Biotech, Guangzhou, China) according to the manufacturer's instructions. The sequences of primers and probe used are as following: 5′-CGTGAGGGCATCGAGGTGGC-3′, 5′-GCGTAGGCGTCGGTGACAAA-3′, and 5′-TGCTACCCACAGCCGGTTAGG-3′, respectively. The result was expressed as log_10_ copies of *Mtb* DNA per ml of sputum.

### Statistical analysis

The sensitivity, specificity, PPV, NPV, LR+, LR- and diagnostic efficiency were calculated using SPSS statistical software (SPSS Inc., Chicago, USA). Positive rates in different groups were compared using χ^2^ test. *P* < 0.05 was considered to be statistically significant by 95% confidence interval analysis. Agreement between tests was assessed by estimating Cohen's κ coefficient, and κ ≥ 0.75 indicates excellent agreement, 0.75 > κ ≥ 0.4 indicates fair to good agreement, and κ < 0.4 indicates poor agreement (Mantegani et al., 2006).

## Results

### Baseline characteristics of study population

Among 307 enrolled participants, 7 were excluded from the final analysis due to unqualified sampling, thus a total of 300 participants were successfully evaluated with both TS-SPOT and T-SPOT.*TB* assays in this study. The sample population included 118 pulmonary tuberculosis (PTB) cases, 37 extra pulmonary tuberculosis (EPTB) cases, 55 healthy controls (HC), and 90 non-TB pulmonary disease patients (Non-TB; Figure 1). Among ATB groups, patients with both EPTB and PTB were classified into the PTB group. The non-TB pulmonary disease patients and health individuals served as negative controls. The baseline characteristics of the enrolled subjects are summarized in Table 1. The mean ages of the patients with PTB, EPTB, Non-TB and healthy controls were 44.29 ± 20.29, 30.24 ± 20.11, 42.62 ± 22.54, and 28.47 ± 6.76, respectively. Male patients were a high proportion in the ATB group. All the participants had been BCG vaccinated. Diabetes was the most common underlying co-morbidity in patients with TB, liver disease, autoimmune diseases, and chronic renal disease followed (Table 1). For 118 PTB cases, apart from those from whom a sputum sample could not be collected and omitting the samples that were contaminated during culturing, 103 sputum smear and 103 bacterial culture results were available for comparison as the “gold standard.”

### Analysis of IGRAs results using clinical diagnosis as reference

Of the 155 clinically diagnosed ATB patients (76.13% PTB and 23.87% EPTB), 124 and 119 cases were detected positively by using the TS-SPOT and T-SPOT.*TB*, respectively, which resulted in the diagnostic sensitivity of 80.00% (124/155) and 76.77% (119/155), respectively. Of the 145 controls including 90 Non-TB pulmonary disease patients and 55 healthy individuals, 24 and 21 cases were detected positively by using the TS-SPOT and T-SPOT.*TB*, respectively, which resulted in the diagnostic specificity of 83.45% (121/145) and 85.52% (124/145), respectively (Table 2). In comparison, the positive rate by smear microscopy was 36.89% (38/103) and was 49.51% (51/103) for bacterial culture. There was a significant difference in diagnostic sensitivity between the bacteriological methods and these two IGRA assays (*p* < 0.01).

**Table 2:** **Comparison of TS-SPOT and T-SPOT.***TB*** assays**.

**Parameter**	**TS-SPOT**		**T-SPOT.*TB***	
	**Positive (*n*)**	**Negative (*n*)**	**Positive (*n*)**	**Negative (*n*)**
ATB Patients (*n* = 155)	124	31	119	36
PTB (*n* = 118)	100	18	96	22
EPTB (*n* = 37)	24	13	23	14
NATB Controls (*n* = 145)	24	121	21	124
Non-TB (*n* = 90)	18	71	17	73
HC (*n* = 55)	6	50	4	51
Sensitivity (%)	80.00		76.77	
Specificity (%)	83.45		85.52	
PPV (%)	83.78		85.00	
NPV (%)	79.61		77.50	
LR+	4.83		5.30	
LR−	0.24		0.27	
Diagnostic efficiency (%)	81.67		81.00	

### Comparison of the performance of TS-SPOT and T-SPOT.*TB* on ATB diagnosis

When using non-TB pulmonary disease patients and healthy individuals as negative controls, the sensitivity and specificity were 80.00 and 83.45% respectively for TS-SPOT, and 76.77 and 85.52% for T-SPOT.*TB*. There was no statistically significant difference between of these assays in either sensitivity or specificity (*P* > 0.05, chi-square test). The PPV of TS-SPOT and T-SPOT.*TB* assays were 83.78 and 85.00%, respectively; the NPV were 79.61 and 77.50%; LR+ were 4.83 and 5.30; LR- were 0.24 and 0.27, and the diagnostic efficiencies for ATB diagnosis were 81.67 and 81.00%, respectively (Table 2). Statistical analysis showed no significant difference between of these two IGRA assays.

### Agreement between TS-SPOT and T-SPOT.*TB* assay

For further comparison of TS-SPOT and T-SPOT.*TB*, the agreement and response correlation between these two assays were analyzed by SPSS 11.0 in the total 300 subjects. Concordance between the assays was measured using the Kappa index, and the correlation between TS-SPOT response and T-SPOT.*TB* response was analyzed non-parametrically by Spearman's correlation. In the 300 subjects, TB-SPOT and T-SPOT.*TB* were both positive in 137 and both negative in 149 subjects, which resulted in an excellent agreement (κ = 0.91, 95% CI: 0.85–0.95) between these two assays (Table 3).

**Table 3:** **Agreement between TS-SPOT and T-SPOT.***TB*** assays**.

**TS-SPOT**	**T-SPOT.*TB***			**Kappa value (95%)**
	**+**	**−**	**Total**	
+	137	11	148	0.91
−	3	149	152	(0.85–0.95)
Total	140	160	300	

**Figure 2:**
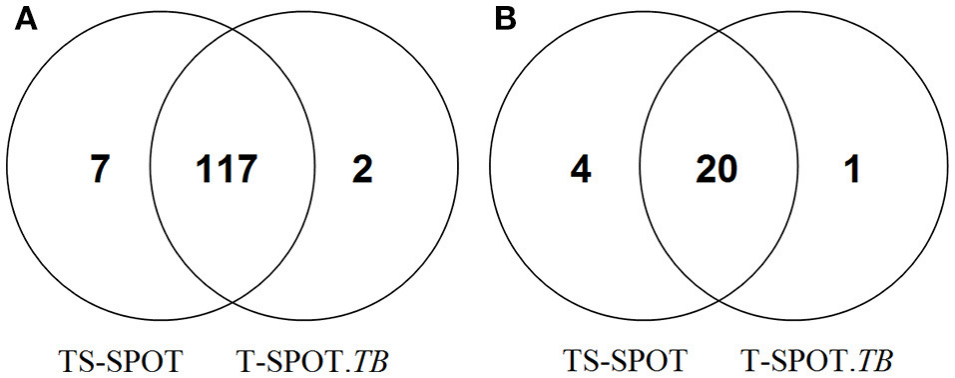
**Positive detection results from the TS-SPOT and T-SPOT.***TB*** assays**. Venn diagram represents the number of positive results from two IGRA assays among the 155 active tuberculosis patients including 118 pulmonary TB and 37 extra-pulmonary TB cases **(A)** and 145 non-TB controls including 90 non-TB pulmonary disease patients and 55 health individuals **(B)**. TS-SPOT and T-SPOT.*TB* were all negative in 29 ATB patients and 120 non-TB control subjects.

Among 155 active tuberculosis patients, 117 subjects were positive by both assays, 9 had discordant results between the two assays: 7 were TS-SPOT^+^/T-SPOT.*TB*^−^ and 2 were TS-SPOT^−^/T-SPOT.*TB*^+^ (Figure 2A). Among 145 controls, 20 were positive by both assays, suggesting that they might be LTBI individuals. Of 145 control subjects, 5 had discordant results between the two assays: 4 were TS-SPOT^+^/T-SPOT.*TB*^−^, 1 was TS-SPOT^−^/T-SPOT.*TB*^+^ (Figure 2B). These data suggest an excellent concordance between these two IGRA assays. The sensitivity of TS-SPOT assay was a little higher than T-SPOT.*TB* for diagnosis of *Mtb* infection including active TB and latent TB infection, although this did not reach statistical significance for either group.

### Performance of TS-SPOT assay for diagnosis of PTB using bacteriological tests as the “gold standard”

**Figure 3:**
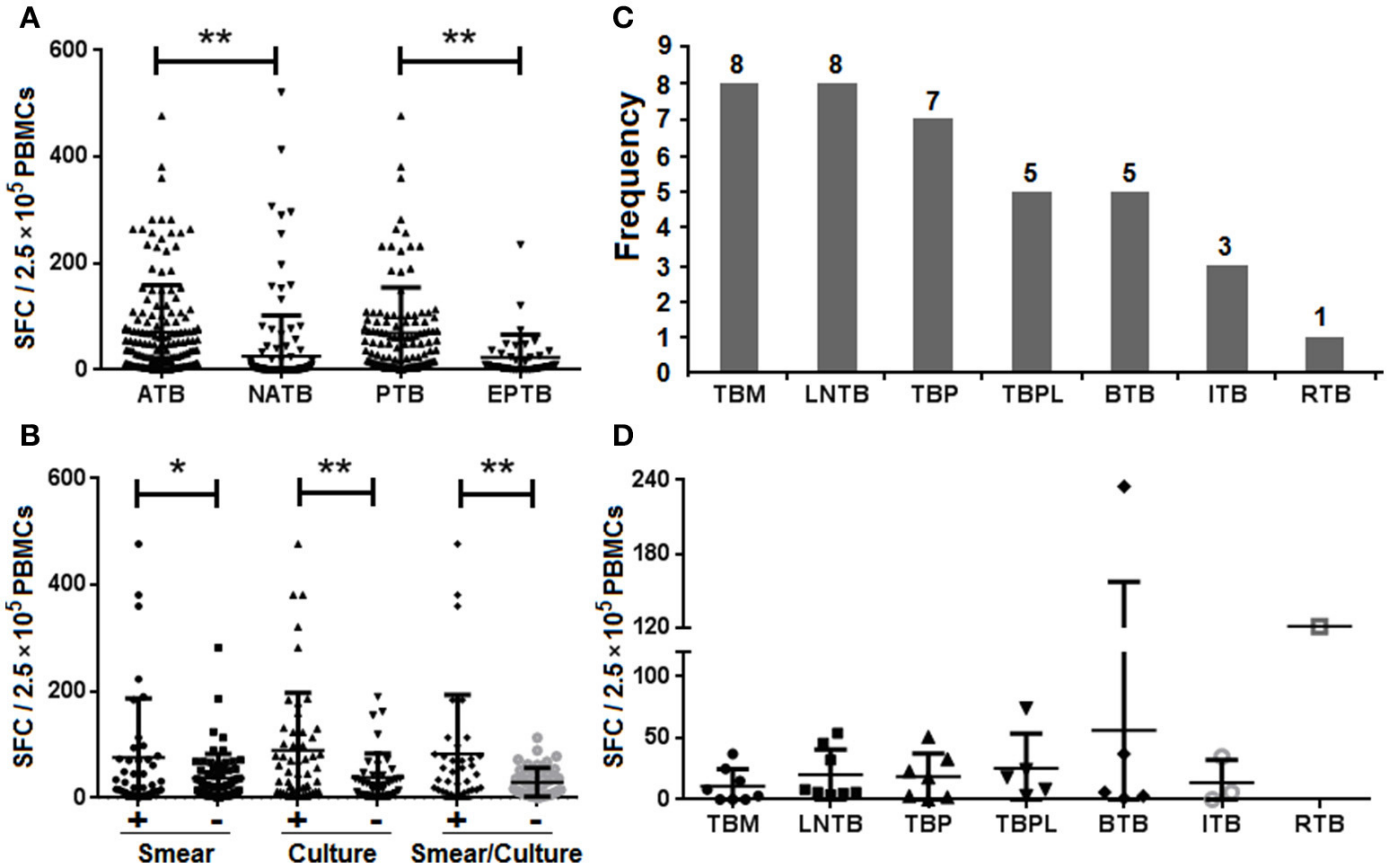
**IFN-γ responses in the different forms of TB by the TS-SPOT assay**. Spot-forming cells (SFCs) in the different groups of ATB, NATB, PTB, and EPTB **(A)**, and in the different PTB subgroups **(B)** are shown. The number of patients **(C)** or SFC numbers **(D)** in the different forms of extra-pulmonary TB cases are also shown. TBM, tuberculous meningitis; LNTB, lymph node tuberculosis; TBP, tuberculous peritonitis; TBPL, tuberculous pleurisy; BTB, bone tuberculosis; ITB, intestinal tuberculosis; RTB, renal tuberculosis. *P < 0.05, **P < 0.01, when compared as indicated.

When the results of bacteriological tests were used as the “gold standard” to further evaluate the performance of the TS-SPOT assay for diagnosis of PTB cases, the SFCs in the bacteria-positive cases were significantly higher than those in the bacteria-negative cases (Figure 3), the sensitivity was 86.84% (33/38) in smear-positive cases, 92.16% (47/51) in culture-positive cases, and 94.28% (33/35) in both smear- and culture-positive cases. These rates were significantly higher than in total ATB cases (80% sensitivity). Additionally, TS-SPOT assay also detected 81.54% (53/65) in smear-negative cases, 73.07% (38/52) in culture-negative cases, and 74.46% (35/47) in both smear- and culture- negative cases (Table 4), which was still significantly higher than detection by sputum smear (36.89%) or bacterial culture (49.51%; Table 4).

**Table 4:** **Detection of PTB cases using bacteriological methods and TS-SPOT assay**.

**TS-SPOT, *n* (%)**	**Smear staining**		**Bacterial culture**		**Total smear + culture**	
	**Positive**	**Negative**	**Positive**	**Negative**	**Positive**	**Negative**
Positive	33 (86.84)	53 (81.54)	47 (92.16)	38 (73.07)	33 (94.28)	35 (74.46)
Negative	5 (13.16)	12 (18.46)	4 (7.84)	14 (26.92)	2 (5.71)	12 (25.53)

### Clinical characteristics associated with false-negative TS-SPOT results in AFB-positive PTB patients

The TS-SPOT assay gave false-negative results for 7 patients with sputum- or culture-positive TB in which included 2 patients with smear- and culture-positive TB. Compared to AFB-positive patients with positive IFN-γ TS-SPOT responses, those with false-negative TS-SPOT results were older (*P* < 0.01), had fewer bacterial DNA copies in sputum specimens measured by real-time PCR (*P* < 0.01), and had proportionally more severe disease although without significant difference, as defined by image examination (Table 5).

**Table 5:** **Clinical characteristics of AFB-positive patients with false-negative TS-SPOT results**.

	**Value for TS-SPOT result**	
	**False negative (*n* = 7)**	**Positive (*n* = 49)**
Age, years (mean± SD)	59.16 ± 14.32^**^	41.69 ± 22.07
<15	0	4
15–65	2	33
>65	5	12
Gender (male: female)	6: 1	32: 17
Immunosuppressive drug (no.)	2	0
Image examination (no. severe: no. mild)	6: 1	19: 30
*Mtb* DNA in sputum (log copies/ml, mean ± SD)	2.93 ± 0.35^**^	4.68 ± 0.89

^*^P < 0.01, when compared with TS-SPOT positive cases in AFB-positive patients.

### IFN-γ responses in relation to the clinical manifestation of TB

It has been suggested that different forms of TB may lead to different immune response profiles and therefore may affect the sensitivity of IGRAs (Nishimura et al., 2008). To address this question, 37 EPTB patients including various numbers of TB meningitis (TBM), lymph node TB (LNTB), TB peritonitis (TBP), TB pleurisy (TBPL), bone TB (BTB), intestinal TB (ITB), and renal TB (RTB; Figure 3C), were recruited and the IFN-γ responses of patients with different forms of TB were compared. In PBMCs from ATB patients the mean magnitude of IFN-γ response to the mixture of *Mtb*-specific antigens (ESAT-6, CFP10 and Rv3615c) was significantly higher than that of PBMCs from NATB controls (Figure 3A). Patients with extra-pulmonary TB had lower IFN-γ responses than those with PTB (Figure 3B), with the highest sensitivity for patients with culture-positive pulmonary TB (47/51, 92.16%) and the lowest for those with tuberculosis meningitis (4/8, 50.00%; Table 4). However, there were no significant differences among the various EPTB forms (Figure 3D). Thus, the positive rate of the TS-SPOT assay in EPTB (24/37, 64.86%) was also lower than that in PTB (100/118, 84.75%).

## Discussion

IGRAs are *in vitro* immunologic diagnostic tests to identify *Mtb* infection. Currently, two systems including QFT-GIT and T-SPOT.*TB* are commercially available, and their use in clinical practice is more and more widespread. Whereas, the IGRAs were initially conceived to support diagnosis of latent infection, an increasing body of evidence is published on its use in detection of active TB infection (Kang et al., 2007; Nishimura et al., 2008; Thomas et al., 2008; Winqvist et al., 2009; Mazurek et al., 2010; Metcalfe et al., 2010). A significant increase in the number of guidelines now includes recommendations for the use of IGRAs in the differential diagnosis of active TB in low-risk TB settings (Denkinger et al., 2011). China is one of the developing countries with high TB prevalence and widespread BCG vaccination, and TST detection has been demonstrated to be of poor specificity due to the cross-reaction with BCG vaccination (Richeldi, 2006). However, while IGRAs have been widely used to detect *Mtb* infections with high specificity and sensitivity, the high risk of *Mtb* infection and high cost of these assays limits their use in clinical practice in China, especially in low-income countryside.

For the first time, a newly licensed PBMCs-based IGRA named TS-SPOT, was used for the diagnosis of active TB, and its performance in detection of *Mtb* infection was compared to that of T-SPOT.*TB* as control in this study. The results showed that the sensitivity, specificity, PPV, NPV, LR+, LR- and diagnostic efficiency on ATB diagnosis of this new assay was 80.00, 83.45, 83.78, 83.45, 4.83, 0.24, and 81.67%, respectively, which were all similar to the imported T-SPOT.*TB* assay that had sensitivity, specificity, PPV, NPV, LR+, LR- and diagnostic efficiency of 76.77, 85.52, 85.00, 85.51, 5.30, 0.27, and 81%, respectively (Table 2). Consequently, there was a nearly complete concordance with an excellent Kappa value of 0.91 between these two PBMCs-based IGRAs, TS-SPOT and T-SPOT.*TB*.

In direct comparison of these two IGRA assays, TS-SPOT detected a higher number of ATB patients than T-SPOT.*TB* (124 vs. 119; Table 2), indicating higher sensitivity of TS-SPOT assay (80.00 vs. 76.77%) for diagnosis of active tuberculosis. The Venn diagrams showed that most of the positive ATB (117/126) cases had consistent results between these two assays. The finding of 7 ATB cases with TS-SPOT^+^/T-SPOT.*TB*^−^ results further indicated that TS-SPOT assay may have a higher sensitivity for diagnosis of active TB than T-SPOT.*TB* (Figure 2A), although there were no statistical differences between them. This may be because of inclusion of the third *Mtb*-specific antigen Rv3615c as stimulus besides ESAT-6 and CFP10 in the TS-SPOT assay; the additional positive cases may be having had unique Rv3615c-specific responses. Similarly, TS-SPOT also showed a higher positive rate in the NATB controls (24 vs. 21) than T-SPOT.*TB* (Table 2) or 4 additional cases with TS-SPOT^+^/T-SPOT.*TB*^−^ results (Figure 2B). This resulted in a lower specificity (83.45 vs. 85.52%) on diagnosis of active tuberculosis when using non-TB patients with pulmonary diseases and healthy individuals as the control population (Table 2). Due to the high prevalence of *Mtb* infection in China, it is plausible that the positive responses of control individuals could be due to LTBI. However, evaluating the accuracy of IGRAs in diagnosing LTBI remains a problem since there is no “gold standard” for such diagnoses. Actually, when analyzing the background information, we note that all of the four additional positive subjects (TS-SPOT^+^/T-SPOT.*TB*^−^) are respiratory physicians/thoracic surgeons or ATB contacts who have no symptoms and are at high risk of infection with tuberculosis. Accordingly, the seeming lower specificity on diagnosis of active TB possibly reflected a higher sensitivity for positive detection of latent TB infection. However, there were also 2 ATB and 1 NATB cases with TS-SPOT^−^/T-SPOT.*TB*^+^ results which remain unexplained.

When further analyzing its performance for diagnosis of pulmonary TB using bacteriological tests as the “gold standard,” TS-SPOT assay showed up to 90% (80/89) agreement in the smear-/culture-positive cases, and 77.78% (91/117) positive rate in the smear-/culture-negative cases, which was significantly higher than the 36.89% (38/103) by sputum smear or 49.51% (51/103) by bacterial culture in the bacteriological tests (Table 4). Certainly, negative results were inevitably seen in 7 patients with sputum- and/or culture-positive TB. Nevertheless, those with false-negative TS-SPOT results were either older or showed fewer bacterial DNA copies compared to AFB- and TS-SPOT-positive patients (Table 5). This was also reported previously from studies using an in-house IFN-γ ELISPOT assay (Chen et al., 2009). Thus, the findings indicate that TS-SPOT assay could be a useful tool for rapid diagnosis of active tuberculosis compared to the low-efficacy of sputum smear and time-consumption of *Mtb* culture.

It should be noted that the sensitivity of IGRAs may vary among different forms of tuberculosis. Consistent with a recent study reporting a lower sensitivity of the T-SPOT.*TB* assay for diagnosis of extra-pulmonary TB compared with PTB (Wang et al., 2015), we also observed a lower magnitude of IFN-γ responses in patients with extra-pulmonary TB than in those with PTB (Figure 3A), which resulted in the higher positive rate in PTB cases (84.75%) than in EPTB cases (64.86%; Table 2). Our results also showed that the magnitude of IFN-γ responses did not differ significantly between EPTB and non-TB controls (Figure 3A) or among the various EPTB forms (Figure 3D). A similar observation was seen in a reported study of Vietnamese patients, which argued against the use of the IFN-γ ELISPOT assay for diagnosis of tuberculous meningitis in blood (Simmons et al., 2006). The low IFN-γ responses seen in patients with severe TB reflects the complex roles of IFN-γ in immunity and the pathogenesis of TB and a comprehensive understanding of the role of IFN-γ responses should lead to better clinical indications, applications, and utilization of IFN-γ as a biomarker (Andersen et al., 2007). Although relatively low IFN-γ responses were seen in the patients with the EPTB forms compared to PTB cases, the positive rate of TS-SPOT in the EPTB cases was still high, up to 64.86% (Table 2). This was significantly higher than the sensitivity (8/37, 21.62%) using the bacteriological tests based on the body fluid samples (Figure 1). Thus, TS-SPOT assay may be a useful adjunct to the current tests for rapid diagnosis of extra-pulmonary TB. The sensitivity might be substantially improved by using body fluid samples of EPTB cases instead of, or in addition to, the blood-based PBMCs; Wang et al. reported that T-SPOT.*TB* showed high sensitivity in the diagnosis of tuberculous pleurisy and peritonitis (82.35 and 80%, respectively) and high specificity (75 and 100%, respectively) when pleural effusion and ascites fluid were used as samples (Wang et al., 2015).

Taken together, the evidence in this current study shows that TS-SPOT, a recently licensed IFN-γ release assay in China, has high sensitivity and specificity in diagnosis of active TB with essentially excellent agreement with the widely available T-SPOT.*TB* assay. Additionally, it may be useful to aid in the clinical detection and diagnosis of *Mtb* infection in patients with smear- and/or culture-negative TB and extra-pulmonary TB. Due to its cost-effective and high-quality characteristics, this assay may be a very useful tool for TB control, especially in low-income and high-incidence settings.

## Author contributions

XYF, JXL, and FL designed the experiments; GL, HMZ, CLL, HLW, HCL, PJ, PX, KW, and ZDH performed the experiments; GL, FL, ZDH, and SHL analyzed the data; XYF and JXL contributed reagents/materials/analysis tools; XYF, GL, and DBL wrote the manuscript.

## Conflict of interest statement

The authors declare that the research was conducted in the absence of any commercial or financial relationships that could be construed as a potential conflict of interest. The reviewer WS and handling Editor declared their shared affiliation, and the handling Editor states that the process nevertheless met the standards of a fair and objective review.
